# Inhibition of the Na^+^-glucose transporter SGLT2 reduces glucose uptake and IFNγ release from activated human CD4^+^ T cells

**DOI:** 10.3389/fimmu.2025.1576216

**Published:** 2025-06-17

**Authors:** Zhe Jin, Hayma Hammoud, Amol K. Bhandage, Stasini Koreli, Azazul Islam Chowdhury, Peter Bergsten, Bryndis Birnir

**Affiliations:** Department of Medical Cell Biology, Uppsala University, Uppsala, Sweden

**Keywords:** glucose uptake, T cells, SGLT2, GLUT1, immunomodulation, IFNγ, empagliflozin, phlorizin

## Abstract

Glucose uptake in activated CD4^+^ T cells is essential for increased metabolic needs, synthesis of biomolecules and proliferation. Although, facilitated glucose transport is the predominant route for glucose entry at the time of activation, here we demonstrate role for the sodium-dependent glucose transporter SGLT2. By 72 h after activation, SGLT2 is expressed and functional in the human CD4^+^ T cells. SGLT2 inhibitors, phlorizin and empagliflozin decreased glucose uptake into the human CD4^+^ T cells compared to untreated cells. Phlorizin (25 μmol/L) reduced glycolysis at 5.6 mmol/L glucose and IFNγ levels at both 5.6 mmol/L and 16.7 mmol/L glucose. In contrast, empagliflozin (0.5 μmol/L) only decreased IFNγ levels in 16.7 mmol/L glucose. GABA enhanced phlorizin inhibition at both 5.6 mmol/L and 16.7 mmol/L glucose in the presence of insulin. Insulin strengthens GABA_A_ receptors signaling in CD4^+^ T cells. The results are consistent with expression of SGLT2 after activation of human CD4^+^ T cells, that facilitates concentrating glucose uptake into the cells, enabling enhanced release of inflammatory molecules like IFNγ. Importantly, inhibition of SGLT2 decreases IFNγ release.

## 1 Introduction

Glucose enters cells by the route of facilitated (GLUTs) (1) or the sodium-dependent (SGLTs) (2) glucose transporters. Glucose is not only an energy source but is also a carbon supplier in the biosynthesis of a variety of biomolecules in cells. The transport is of vital importance for growth and survival of cells. In CD4^+^ T cells, glucose transport is essential for the rapid proliferation and differentiation of the activated cells (3, 4). From the time of activation, glucose is transported by facilitated glucose transporters that equilibrate glucose across the cell membrane (1). Activated CD4^+^ T cells shift their metabolic phenotype to aerobic glycolysis at the expense of oxidative phosphorylation (5) but for the advantage of biosynthetic pathways that branch out from the glycolysis pathway (6). Hexokinase is the first enzyme in the glycolytic pathway and catalyzes the reaction that converts glucose to glucose-6-phosphate (G-6-P) that effectively traps glucose within the cell (7). The neurotransmitter γ-aminobutyric acid (GABA) down-regulates hexokinase in human CD4^+^ T cells (8). Hexokinase activity is then regulated by a number of molecules including feedback inhibition by G-6-P (7). Still, this inhibition is competitively decreased by high intracellular glucose concentrations (7). In humans, the normal fasting physiological blood glucose concentration ranges from 4 to 5.6 mmol/L but it can be altered under pathophysiological conditions, as is observed in diabetes-related hypoglycemia (< 3 mmol/L) or hyperglycemia (> 11 mmol/L) (9). In T cells, the insulin receptor is expressed from 48 h after activation (10). In T cells, activation of the insulin receptor leads to enhanced GABA_A_ receptors signaling (8).The facilitated glucose transporters (GLUTs) expressed in human T cells have glucose affinity between 1–3 mmol/L glucose that renders them constitutively active at normal physiological glucose concentrations (1, 11, 12). Whether the nature of glucose transport alters in T cells when glucose levels are elevated or with time after activation of the CD4^+^ cells has not been established to-date.

Here we show in human CD4^+^ T cells, 72 h after activation, that the sodium-dependent glucose transporter SGLT2 is expressed, functional and contributed significantly to glucose uptake. The SGLT2s’ inhibitors phlorizin and empagliflozin significantly reduced glucose uptake into cells when grown in either normal (5.6 mmol/L) or high (16.7 mmol/L) glucose concentration. The inhibitors alone decreased IFNγ secretion and phlorizin enhanced the GABA plus insulin inhibition of IFNγ release, supporting synergistic effects of GABA and the SGLT2 inhibitors. The results imply that SGLT2 inhibitors in humans, in addition to decreasing blood glucose, improving metabolic control and providing cardiac and renal protection (13, 14), also decrease CD4^+^ T cell-mediated inflammation.

## 2 Materials and methods

### 2.1 Study individuals and collection of samples

Human blood samples were obtained from 43 healthy individuals, 22 women and 21 men with the mean age of 26.1 ± 0.5 years, at Uppsala University Academic Hospital under a protocol approved (dnr 2013/347) by the Regional Research Ethical Committee in Uppsala. All subjects were voluntarily recruited and informed consent were provided and signed.

### 2.2 Chemicals and reagents

Drugs, buffers, and salts, unless otherwise stated, were obtained from Sigma-Aldrich/Merck (Steinheim, Germany). WZB 117 were purchased from Tocris (UK, 7A/109031). Phlorizin and empagliflozin were obtained from AdooQ Bioscience.

### 2.3 CD4^+^ T cells isolation and activation

Peripheral blood mononuclear cells (PBMCs) were isolated from blood by density-gradient centrifugation using Ficoll-Paque™ PLUS (Sigma Aldrich, Sweden). CD4^+^ T cells were then purified from the isolated PBMCs using negative selection with human CD4^+^ T cell isolation kits (Miltenyi Biotec, Germany), following the manufacturer’s instructions. Freshly isolated CD4^+^ T cells (1x 10^6^ cells/ml) were cultured in RPMI 1640 glucose-free medium (Gibco, Fisher Scientific, Sweden) supplemented with 2 mmol/L glutamine, 10% heat-inactivated dialyzed fetal bovine serum (FBS), 100 U/ml penicillin, 10 mg/ml streptomycin, and 5 μmol/L β-mercaptoethanol (Gibco, Fisher Scientific, Sweden). CD4^+^ T cells were activated with 3 μg/ml plate-bound anti-CD3 antibody (BD Biosciences, USA, 555329), in either 5.6 mmol/L or 16.7 mmol/Lglucose for 72 h in either 96-well (10^5^ cells/well) or 24-well (10^6^ cells/well) plates. Jacobs et al. using similar anti-CD3 concentration (5 μg/ml) as employed in this study (15) demonstrated that when anti-CD3 alone is used, the proliferation of the T cells is about 1/3 of the maximal stimulation obtained when co-stimulated with anti-CD28 at the maximal concentration of anti-CD3 (5 μg/ml). Insulin added at 48 h after activation stimulates activation of Akt dependent-signaling, similar to the anti-CD28 activation (15). Experimental drugs were added based on the experimental design and identified in figures and figure legends.

### 2.4 MTT assay

Metabolic activity of CD4^+^ T cells was assessed using a colorimetric MTT (3-(4,5-Dimethylthiazol-2-yl)-2,5-Diphenyltetrazolium Bromide) assay in 96-well plates. After 72 h of activation, a solution of MTT (final concentration 1 mmol/L) in PBS was added and incubated for an additional 4 h at 37°C. Cells were then centrifuged at 2000 rpm for 10 min to concentrate the insoluble purple formazan crystals. Supernatants were collected and stored at −80°C for subsequent IFNγ analysis. The formazan crystal crystals were dissolved in DMSO, and absorbance was measured 10 min later using a Multiskan FC Microplate Photometer (Thermo Fisher, USA) at 550 nm.

### 2.5 Trypan blue exclusion test of cell viability

CD4^+^ T cells 72 h post-activation were washed, resuspended in 1x PBS, and mixed with 0.4% trypan blue (Sigma-Adrich, Germany). A portion of the cell/trypan blue mixture was loaded into a Burker counting chamber. Viable (unstained) and nonviable (stained) cells were counted separately, and the percentage of viable cells under drug treatment was calculated.

### 2.6 Total RNA and bulk RNA sequencing

Total RNA was extracted from activated CD4^+^ T cells using RNA Purification Plus Kit (Norgen Biotek, Ontario, Canada). During the extraction process, total RNA was treated with RNase-Free DNase I Kit (Norgen Biotek, Ontario, Canada) to eliminate contamination of genomic DNA. Total RNA samples were submitted to the GENEWIZ NGS facility (Germany) and quality control analysis showed that all RNA samples had an RNA integrity number (RIN) >9. Strand-specific RNAseq cDNA libraries with polyA selection were generated and sequenced using Illumina HiSeq sequencing platform. Sequence reads were filtered and trimmed using Trimmomatic V.0.36 to remove low quality reads. The trimmed reads were further mapped and aligned to the Homo Sapiens GRCh38 reference genome (ENSEMBL) using the STAR aligner v.2.5.2b. Unique gene hit counts were calculated using feature Counts from the Subread package v.1.5.2. RNAseq data were deposited in the Gene Expression Omnibus (GEO) database (GSE230041).

### 2.7 Western blot analysis

CD4^+^ T cells were lysed in RIPA buffer (Sigma-Aldrich, R0278) supplemented with a protease inhibitor cocktail (Roche, 11 836 153 001). Lysates were denatured by heating at 95°C for 5 min in SDS loading buffer (275 mmol/L Tris. HCL, 9% SDS, 50% glycerol, 0.03% bromophenol blue) containing 9% β-mercaptoethanol. Proteins were separated by SDS-PAGE using 4-15% Mini-Protean TGX stain-free gels (BioRad, #4568084) and transferred to Amersham Hybond-P PVDF membrane. Membranes were blocked with EveryBlot blocking buffer (BioRad, 12010020) for 20 min at room temperature (RT) and then incubated overnight at 4°C with a rabbit polyclonal anti-SGLT2 antibody (ProteinTech, 24654-1-AP, 1:500). Following primary antibody incubation, membranes were washed with TBS-T and incubated with a peroxidase-conjugated AffiniPure secondary antibody (Jackson ImmunoResearch, 111-035-045, 1:5000) for 1 h at RT. Protein bands were visualized using ECL chemiluminescent reagent (Thermo scientific, 32209) and quantified with Image Labs software (BioRad Laboratories, V6.0.0).

### 2.8 Immunofluorescence staining

CD4^+^ T cells were collected after 72 h of anti-CD3 activation and spun onto cytoslides (Thermo scientific, 5991056) using a Shandon Cytospin centrifuge at 400 rpm for 4 min at RT. Following air-drying, the cells were fixed with 4% PFA in PBS for 15 min, followed by three PBS washes and by membrane permeabilization with 0.1% Triton-X for 5 min. Non-specific binding was blocked by incubating the slides in 5% bovine serum albumin (BSA) and 10% donkey serum (Jackson ImmunoResearch, 017-000121) in PBS for 60 min. The cells were then washed and incubated overnight at 4°C with a rabbit polyclonal anti-SGLT2 antibody (ProteinTech, 24654-1-AP, 1:100) diluted in blocking solution. After incubation, the cells were washed with PBS and further incubated with an AlexaFluor 488-conjugated AffiniPure Donkey Anti-Rabbit IgG secondary antibody (Jackson ImmunoResearch, 711-545-152, 1:500) for 1h at RT. Finally, the cells were washed again, counterstained with DAPI for 5 min. Slides were mounted using ProLongTM Gold antifade reagent (Invitrogen), and images were acquired using a Zeiss LSM700 confocal microscope (Zeiss) with a 63X objective.

### 2.9 Glucose uptake assay

A glucose uptake assay was performed using 2x 10^6^ CD4^+^ T cells that had been activated for 72 h, with insulin treatment initiated 48 h post-activation. The cells were centrifuged, resuspended in glucose-free RPMI-1640 media, incubated for 10 min at 37°C, and washed once with the same media. Subsequently, glucose-free media (500 μl) containing 1μCi/ml [^14^C] glucose (Perkin Elmer) were added to the cells, either in the absence or presence of phlorizin (100 μmol/L) or empagliflozin (0.5 μmol/L). The cells were incubated for 40 min at 37°C, centrifuged at 300 x g for 5 min, and washed three times in cold PBS. Following this, the cells were lysed in RIPA buffer, and the ^14^C content was quantified using Gold XR scintillation fluid in a liquid scintillation counter (Perkin Elmer). Glucose uptake was determined by normalizing radioactivity counts to the protein content of each sample.

### 2.10 Metabolic flux analysis

The metabolic activity of activated CD4^+^ T cells was assessed using a Seahorse XF^e^96 Extracellular Flux Analyzer (Seahorse Bioscience). CD4^+^ T cells were activated for 66–72 h *in vitro* and then plated onto Cell-Tak (Corning, New York, USA, #354240) coated XF^e^96 cell culture plates according to manufacturer’s protocol. The extracellular acidification rate (ECAR), a measure of glycolytic activity, was determined using the glycolysis stress test protocol. Briefly, cells were maintained in XF media (Seahorse Bioscience) supplemented with 5 mmol/L glucose (Sigma Aldrich). Following baseline ECAR measurements, cells were sequentially treated with 25 mmol/L glucose, 2 μmol/L oligomycin, and 15 mmol/L 2-deoxyglucose (2DG). Measurements were performed at least in triplicate. The calculated parameters of glycolysis, glycolytic capacity, and glycolytic reserve were then normalized to the protein content per well.

### 2.11 IFNγ ELISA analysis

IFNγ concentrations in cell supernatants were measured using commercially available ELISA kits (Human IFNγ DuoSet ELISA, R&D Systems, USA and ELISA MAX Deluxe Set Human IFNγ, BioLegend, UK) following the manufacturers’ instructions. The supernatants were diluted as needed to fall within the linear optical range of the assay. Optical densities were read at 450 nm, with a correction wavelength of 540 nm, using an FC Microplate Photometer (Thermo Fisher, USA).

### 2.12 Statistical analysis

Statistical analysis was conducted using GraphPad Prism 9 (GraphPad Software Inc., La Jolla, CA, Version 9.3.1). Data normality was assessed using the Shapiro-Wilk test. For donor-matched data, a two-tailed paired Student’s t-test (for normally distributed data) or the Wilcoxon matched-pairs signed rank test (for non-normally distributed data) was applied. Normalized data compared to controls were analyzed using a one-sample t-test. For comparisons between two independent groups, an unpaired Student’s t-test was used for normally distributed data. Data were presented as individual values and as mean ± SEM. Statistical significance was defined as P<0.05.

## 3 Results

### 3.1 The SGLT2 is expressed in activated human CD4^+^ T cells

**Figure 1:**
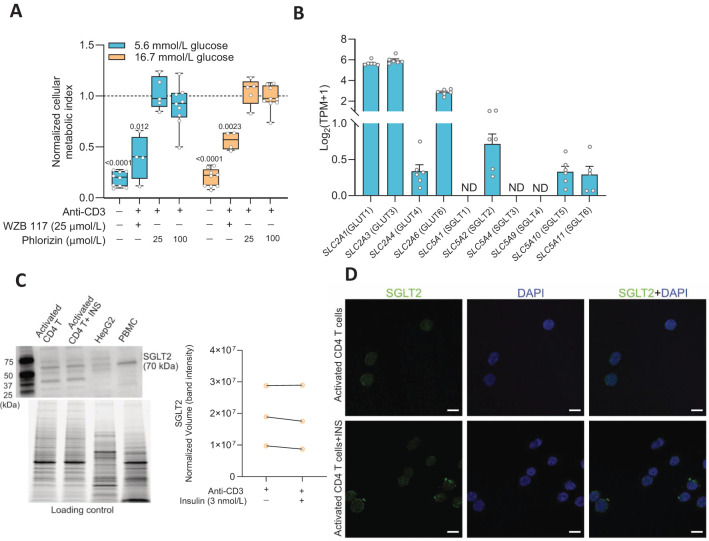
Sodium-glucose co-transporter 2 (SGLT2) is expressed in activated human CD4^+^ T cell. **(A)** The cellular metabolic activity of resting and activated cells in presence of GLUT1 inhibitor, WZB 117 or SGLTs inhibitor, phlorizin was normalized to that of activated CD4^+^ T cells without drug treatment in 5.6 mmol/L or 16.7 mmol/L glucose (N= 4-9). Data are presented as box and whisker plot: box as 25–75 percentiles, whiskers determined with Tukey’s method, black lines in the boxes as median. Statistics: one sample t-test when compared to activated cell group. **(B)** Relative gene expression levels of facilitated glucose transporters (GLUTs) and Na^+^-dependent glucose transporters (SGLTs) in activated CD4^+^ T cells at 5.6 mmol/L glucose (N=7) determined by RNAseq analysis. TPM, transcripts per million. Data represent mean ± SEM. ND, not detected. **(C)** Representative Western blot image of SGLT2 protein in activated CD4^+^ T cells in the absence or presence of insulin (INS, 3 nmol/L) at 16.7 mmol/L glucose, HEPG2 cell line and peripheral blood mononuclear cell (PBMC). The stain-free blot image below represents total proteins loaded for each sample. Quantification of SGLT2 band intensity as normalized volume value, which represents each chemiluminescent signal (band) that is properly total protein normalized to the stain-free signal in its corresponding lane (N=3, 16.7 mmol/L glucose). **(D)** Representative immunofluorescence co-staining of SGLT2 (green) and DAPI (blue) in activated CD4^+^ T cells in the absence or presence of insulin (INS, 3 nmol/L) at 16.7 mmol/L glucose. Scale bar: 10 µm. N, number of donors.

Facilitated glucose transport is the main route for glucose entry into activated T cells (1) but as the cells enlarge, multiply and environmental factors change, other types of glucose transporters might become expressed. We examined the effects of glucose transporters inhibitors on the cellular metabolic activity at 72 h after activation of the CD4^+^ T cells. We applied WZB117 (25 μmol/L), a GLUT inhibitor, or phlorizin (25 or 100 μmol/L) (16), an inhibitor of SGLT1 and SGLT2, to the CD4^+^ T cells and the results are shown in **Figure 1A**. WZB117 decreased whereas phlorizin had no effect on the metabolic activity of the CD4^+^ T cells when they were grown in either 5.6 mmol/L or 16.7 mmol/L glucose concentration (**Figure 1A**). None of the drugs affected the viability of the cells when measured at 72 h after activation (**Supplementary Figure 1A**). We then examined gene expression 72 h after activation of the transporters using RNAseq analysis (**Figure 1B**). Several facilitated GLUTs gene transcripts (e.g., *SLC2A1* and *SLC2A3*) were prominently expressed in the activated CD4^+^ T cells but, in addition, *SLC5A2*, the gene encoding the sodium-dependent glucose transporter SGLT2, was also detected albeit at a modest level (**Figure 1B**). Western blot and immunofluorescence staining data then demonstrated SGLT2 protein expression in the activated CD4^+^ T cells (**Figures 1C, D**). A question that remained was if the SGLT2 transporter protein formed functional glucose transporters that were transported to and inserted into the plasma membrane of the CD4^+^ T cells.

### 3.2 The SGLT2 inhibitors phlorizin and empagliflozin reduce glucose uptake into activated human CD4^+^ T cells

**Figure 2:**
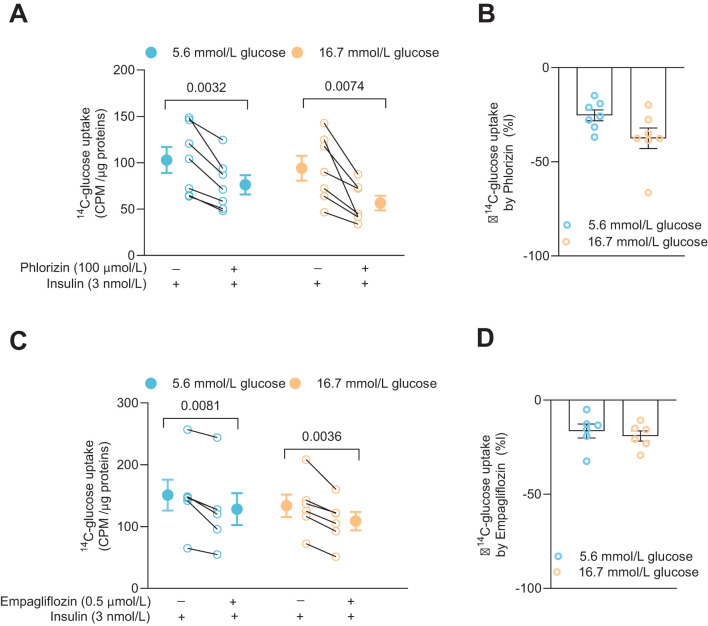
SGLT inhibitors reduce glucose uptake in activated human CD4^+^ T cell. Glucose uptake in absence or presence of phlorizin (**A**, N=7) and SGLT2 selective inhibitor, empagliflozin (**C**, N=6) in cells 72 h post-activation, with insulin, in 5.6 mmol/L or 16.7 mmol/L glucose. The changes in percentage of glucose uptake by phlorizin and empagliflozin are shown in **(B, D)**, respectively. Data are presented as individual values and mean ± SEM. Statistics: two-tailed paired Student’s t-test **(A, C)** and unpaired Student’s t-test **(B, D)**. CPM, count per minute. N, number of donors.

Glucose uptake and the effects of SGLT2 inhibitors were examined in the CD4^+^ T cells 72 h after activation. The cells had been cultured together with insulin (3 nmol/L) at either 5.6 mmol/L or 16.7 mmol/L glucose. Phlorizin (100 μmol/L) or empagliflozin (0.5 μmol/L), a prescription drug used to treat type 2 diabetes, were applied to the cells in the presence of radiolabeled glucose. Both phlorizin (**Figure 2A**) and empagliflozin (**Figure 2C**) significantly reduced the glucose uptake. Phlorizin was about two times more effective than empagliflozin decreasing the glucose uptake (**Figures 2B, D**). The difference in the effectiveness of the inhibitors may be related to their concentrations where phlorizin (Ki = 11 nmol/L in 5 mmol/L glucose (16)) was present at supersaturating concentration whereas empagliflozin concentration (Kd = 57 nmol/L at 0 mmol/L glucose, 194 nmol/L at 20 mmol/L glucose) (17)) was 200 times lower. Somewhat surprisingly, cells grown in 5.6 mmol/L or 16.7 mmol/L glucose had similar tracer glucose uptake and the uptake was also similarly reduced at both glucose concentrations for each inhibitor, indicating that the glucose concentration in the extracellular milieu did not alter the functional expression of the glucose transporters in the human CD4^+^ T cells (**Figures 2B, D**).

### 3.3 Phlorizin decreases glycolysis in activated human CD4^+^ T cells

**Figure 3:**
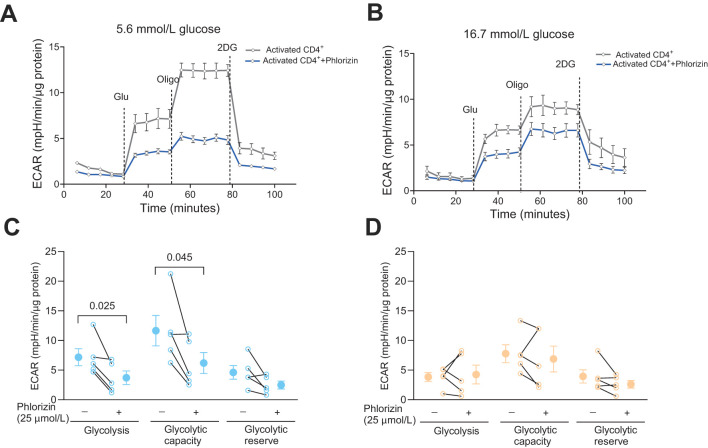
The effect of SGLT inhibitor on glycolysis in activated CD4^+^ T cells. Seahorse analysis of extracellular acidification rate (ECAR) **(A, B)** and glycolysis, glycolytic capacity and glycolytic reserve **(C, D)** in activated cells, 72 h post-activation, incubated without or with phlorizin, in 5.6 mmol/L **(A, C)** or 16.7 mmol/L **(B, D)** glucose. Data are representative of 5 independent experiments and are expressed as individual values **(C, D)** and mean ± SEM. Statistics: two-tailed paired Student’s t-test. N, number of donors.

We have previously reported that the total glycolytic capacity is reduced in human CD4^+^ T cells cultured with high 16.7 mmol/L glucose relative to cells cultured with the normal 5.6 mmol/L glucose level, despite that only low level of exhaustion was recorded for the cells (8). We, therefore, next examined the effect of phlorizin (25 μmol/L) on glycolysis in the CD4^+^ T cells (**Figure 3**). In 5.6 mmol/L glucose, phlorizin significantly inhibited glycolysis and the glycolytic capacity (**Figures 3A, C**) whereas in 16.7 mmol/L glucose (**Figures 3B, D**), neither glycolysis nor the glycolytic capacity was consistently reduced by phlorizin. The decrease in the glycolytic capacity and the apparent decrease of phlorizin inhibition for CD4^+^ T cells cultured at 16.7 mmol/L glucose, enticed us to examine the effects of the SGLT2 inhibitors on release of IFNγ.

### 3.4 The SGLT2 inhibitors reduce IFNγ release from activated human CD4^+^ T cells

**Figure 4:**
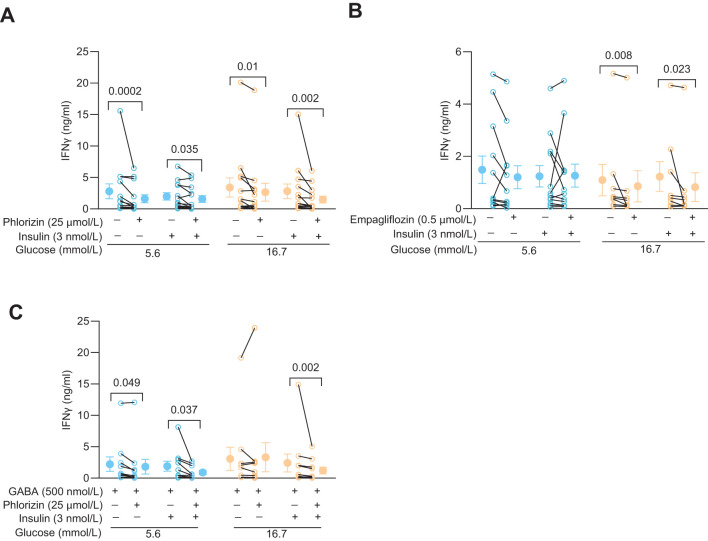
SGLT2 inhibitors and GABA modulate IFNy release in activated human CD4^+^ T cells. **(A, B)** IFNγ release from 72 h post-activated cells 72 h post-activation treated with or without phlorizin (**A,** N=13), empagliflozin (**B,** N=12 at 5.6 mmol/L and N=8 at 16.7 mmol/L glucose) in the absence or presence of insulin, at 5.6 mmol/L or 16.7 mmol/L glucose. **(C)** IFNγ release from 72 h post-activated cells treated with or without phlorizin or insulin in the presence of GABA (N=10), at 5.6 mmol/L or 16.7 mmol/L glucose. Data are presented as individual values and mean ± SEM. Statistics: Wilcoxon matched-pairs signed rank test. N, number of donors.

Since phlorizin reduced glucose uptake and glycolysis, at least at 5.6 mmol/L glucose, it might also decrease synthesis of biomolecules such as IFNγ. **Figure 4** shows effects of phlorizin (25 μmol/L, **Figure 4A**) and empagliflozin (0.5 μmol/L, **Figure 4B**, 25 μmol/L, **Supplementary Figure 1B**) on the levels of IFNγ released from activated CD4^+^ T cells. Indeed, at both 5.6 mmol/L and 16.7 mmol/L glucose concentrations, in the presence or absence of insulin, phlorizin reduced release of IFNγ up to 40% (**Figure 4A**). Similarly, empagliflozin decreased the release of IFNγ but only consistently in 16.7 mmol/L glucose (**Figure 4B**, **Supplementary Figure 1B**). IFNγ levels are not only regulated by glucose uptake, but also by the quantity and activity of the enzymes of the glycolytic pathway (5, 8). GABA decreases the protein level of hexokinase 1 (8), the gatekeeping enzyme of the glycolytic pathway, and inhibits IFNγ release from CD4^+^ T cells in 5.6 but not in 16.7 mmol/L glucose (8). Both, SGLT2 inhibitors and GABA, clearly modulate IFNγ release albeit by different mechanisms. In order to study if these different mechanisms were additive, we examined if the effects of GABA and phlorizin converged and reduced the IFNγ level more than GABA alone (**Figure 4C**). In the presence of insulin, phlorizin plus GABA decreased the IFNγ levels as compared to GABA alone, at both 5.6 mmol/L and 16.7 mmol/L glucose concentrations. In contrast, in the absence of insulin, and only at 5.6 mmol/L glucose, GABA and phlorizin together enhanced the inhibition of IFNγ release. The levels of IFNγ were similar at 16.7 mmol/L glucose in GABA alone or together with phlorizin. These results suggest that the strengthening of GABA signaling by insulin (8) is required to achieve inhibition in combination with phlorizin at the high 16.7 mmol/L glucose concentration. Furthermore, at 16.7 mmol/L glucose, phlorizin failed to inhibit when combined with GABA in the absence of insulin, unlike its inhibitory effect when used alone (**Figure 4A**). This finding is somewhat reminiscent of empagliflozin’s lack of inhibition in 5.6 mmol/L glucose, although empagliflozin did reduce IFNγ levels at the higher 16.7 mmol/L glucose concentration. The results support a dynamic regulation of INFγ release by intracellular glucose levels and the concentration and activity of enzymes of the glycolytic pathway.

## 4 Discussion

**Figure 5:**
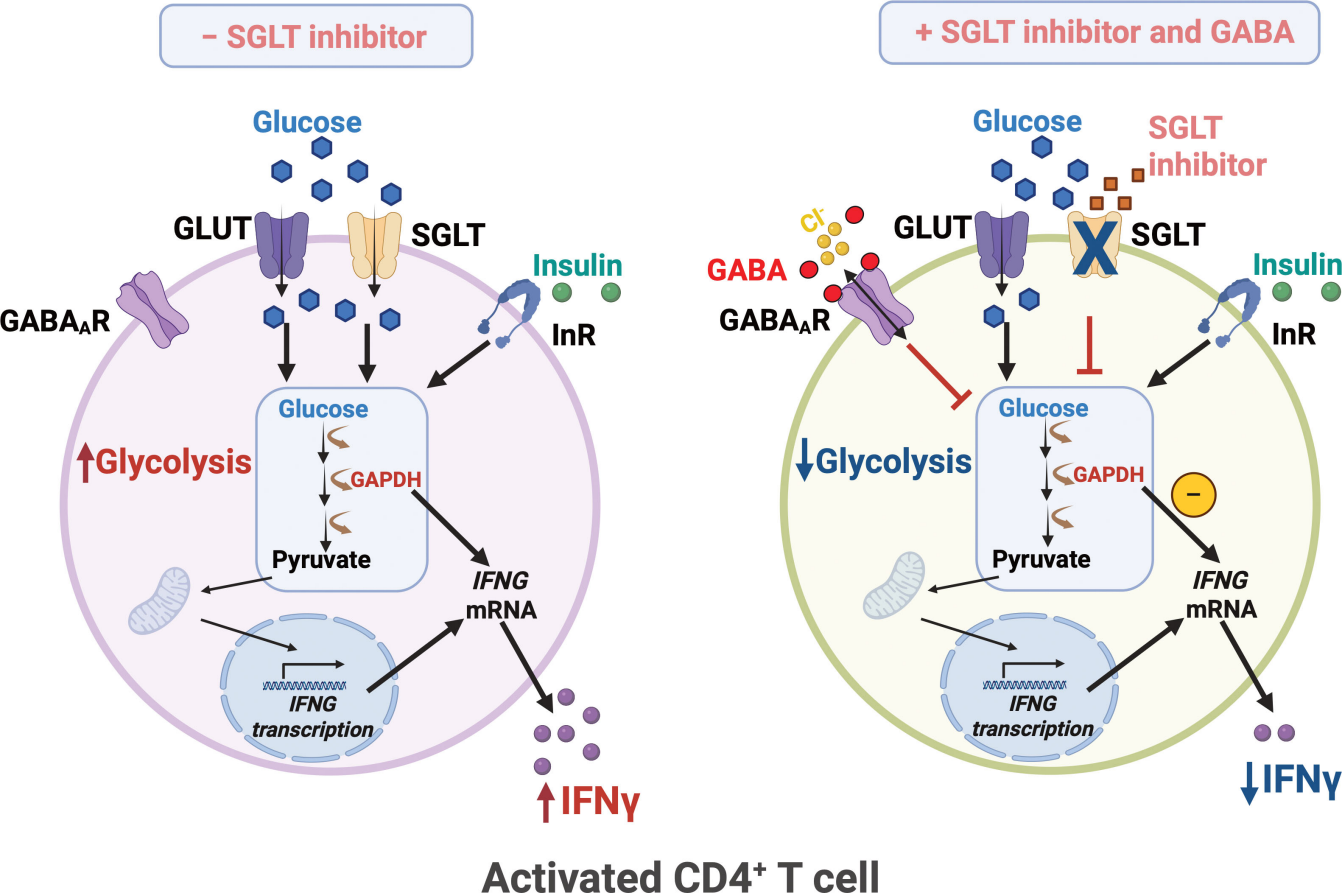
SGLT inhibitor and GABA regulate glycolysis and IFNy release in human CD4^+^ T cells. Upon activation, human CD4^+^ T exhibit enhanced glycolysis and increased IFNγ release. Glucose uptake is mediated by not only the facilitated glucose transporters GLUT1/3 but also the sodium-dependent glucose transporter 2 (SGLT2). SGLT inhibitor together with GABA, attenuates glycolysis and reduces IFNγ levels. InR, insulin receptor; *IFNG*, IFNγ gene. Created in BioRender. Jin, Z. (2025) https://BioRender.com/z20f028.

Glucose transport into activated CD4^+^ T cells is predominantly mediated by facilitated glucose transporters (1) and sodium-dependent glucose transporters have not been thought critical for normal CD4^+^ T cells functions (1, 18, 19). Yet the present study identified significant SGLT2 transport by 72 h after activation of the CD4^+^ T cells (**Figure 5**). Both the SGLT2 inhibitors phlorizin and empagliflozin reduced uptake of glucose into the cells at normal (5.6 mmol/L) and high (16.7 mmol/L) glucose concentrations and, decreased IFNγ release from the activated human CD4^+^ T cells. In the presence of insulin, co-application of phlorizin and GABA enhanced the GABA inhibition at both glucose concentrations. The synergistic inhibition of IFNγ release by phlorizin and GABA reveals a mechanism for decreasing human CD4^+^ T cell-induced inflammation associated with normal but also elevated glucose concentrations.

In resting state naïve CD4^+^ T cells have low energy needs but when activated, their energy requirement is greatly increased and required to enable the active metabolic state, proliferation and biosynthesis (1, 5). Activated CD4^+^ T cells switch to aerobic glycolysis, a phenomenon called the Warburg effect (5), where the cells generate ATP and accumulate biomass (5, 6). The family of facilitated glucose transporters supports the increased glucose uptake during the CD4^+^ T cell activation (1). The rate limiting step of the glucose metabolism is glucose transport by GLUT1 (1). Sodium-dependent glucose transport i.e. the SGLT family of transporters, has commonly thought to be absent in CD4^+^ T cells (1) but a few recent studies have identified SGLTs in CD4^+^ T cells (18, 20). It appears clear that the GLUTs are the primary glucose transporters during activation and proliferation of T cells from healthy individuals. However, the glucose affinity of the most prominent facilitated transporters, the GLUT1 (and GLUT3) is 1–3 mmol/L glucose, a fair bit below the normal physiological glucose concentration in blood. These transporters are low-capacity transporters saturated at concentrations between 5–10 mmol/L (11, 12). Thus, in order to increase glucose transport via the GLUTs, it is necessary to increase the density of the transporters in the membrane. On the other hand, SGLT2 is a low affinity, EC_50_ about 5 mmol/L glucose, but high capacity and can, in addition, concentrate the glucose within the cells. At 16 mM glucose, the SGLT2 is about 80% saturated (2, 16). SGLT2 is thus far more effective bringing glucose into the cells at high glucose concentrations than the GLUTs but, perhaps, similar at physiological concentrations where the GLUTs are saturating and working at full capacity. Having dissimilar mechanisms that allow uptake at normal physiological glucose concentrations but that cover the different extremes i.e. very low or very high glucose concentrations, is not uncommon in biology and may be advantageous for the organism. Our results demonstrate that at 72 h after activation, both GLUTs and SGLT2 are expressed in the human CD4^+^ T cells. The SGLT2 will be effective at transporting glucose into the cells at both increasing extracellular as well as increasing intracellular glucose concentration (16), at both normal (5.6 mmol/L) and high (16.7 mmol/L) extracellular glucose. Importantly for CD4^+^ T cells, the transport activity of these cotransporters will increase the intracellular availability of glucose, beyond what is possible with the facilitated glucose transporters, such as GLUT1 (EC_50_ = 3 mmol/L) (1, 12) or GLUT3 (EC_50_ = 1.4 mmol/L) (1), which are also expressed in these cells. It is possible that expression of these transporters is increased in high glucose but since we saw no difference in ^14^C-glucose tracer uptake into cells grown in either 5.6 or 16.7 mmol/L glucose, this is unlikely. The different properties of the transporters are relevant for the function of the CD4^+^ T cells, since only SGLT2s can concentrate glucose intracellularly at both normal and high glucose concentration and, thereby, overcome the rate-limiting effect of glucose transport for the cellular metabolism and biosynthesis.

Hexokinase is the first enzyme in the glycolytic pathway. It converts glucose to glucose-6-phosphate (G-6-P) and traps glucose within the cell (7). The human hexokinase normally has high activity in 5 mmol/L glucose in the presence of natural inhibitors, such as G-6-P (7). Yet, hexokinase activity is increased at glucose concentrations > 5 mmol/L resulting in increased availability of intermediate biomolecules (7). We have previously reported that the neurotransmitter GABA down-regulates the hexokinase protein in CD4^+^ T cells (8). GABA also promoted a glycolytic switch to lower values for glycolysis in 5.6 mmol/L glucose but this switch was not observed in 16.7 mmol/L glucose (8). Similarly, the SGLT2 inhibitor phlorizin only consistently inhibited glycolysis in 5.6 mmol/L glucose, although it inhibited glucose uptake at both 5.6 mmol/L and 16.7 mmol/L glucose as well as inhibited IFNγ release at both glucose concentrations. During aerobic glycolysis, inhibition by GAPDH of the IFNγ mRNA translation is released, as the enzyme is now engaged in the glycolytic pathway (5). Although GABA down-regulated the hexokinase protein, it only decreased hexokinase activity and the IFNγ level at 5.6 mmol/L glucose, indicating the GABA inhibition was overcome by high intracellular glucose in 16.7 mmol/L glucose (8). In accordance, here we demonstrated that the GABA activation of GABA_A_ receptors together with SGLT2 inhibition significantly reduced the levels of IFNγ, not only at normal but also at the high 16.7 mmol/L glucose concentration. The results suggest convergence and additive effects of the GABA-activated inhibition of hexokinase together with the phlorizin inhibition of SGLT2. That IFNγ production varies and is regulated at the level of protein concentration and activity of enzymes of the glycolytic pathway plus the availability of intracellular glucose, enables a wide dynamic range for IFNγ biosynthesis and regulation.

Empagliflozin is an inhibitor at SGLT2 and is used to treat diabetes by lowering blood glucose levels by inhibiting reabsorption in the kidneys (21, 22). It is a potent, competitive SGLT2 inhibitor, with Kd of 194 nmol/L in 20 mmol/L glucose (17). In addition, an off-target has been identified in heart (23). Empagliflozin (0.5 μmol/L) inhibited glucose uptake into the CD4^+^ T cells about 20%, which is lower inhibition than by phlorizin but may be related to the concentrations used. Somewhat surprisingly, IFNγ release was only decreased in the high 16.7 mmol/L glucose by empagliflozin. It is possible that this reflects the relative inhibition on the intracellular substrate availability for the glycolytic pathway. Phlorizin, decreased IFNγ levels at both glucose concentrations but also exhibited stronger inhibition on glucose uptake. The current results are consistent with empagliflozin modulating levels of inflammatory molecules (20, 22, 24) and may potentially lower inflammation in diabetes by decreasing glucose uptake into CD4^+^ T cells, resulting in e.g. decreased IFNγ release from the cells.

In this study we have examined effects of SGLT2 inhibitors on functional properties of CD4^+^ T cells from healthy donors (**Figure 5**). A limitation of the study is that we do not study SGLT2 function in CD4^+^ T cells from diabetic patients and how SGLT2 inhibitors modulate glucose uptake in these cells. Furthermore, we have only studied the effects of SGLT2 inhibitors on IFNγ secretion. Clearly, it would be informative to examine how SGLT2 inhibitors affect the levels of other inflammatory biomolecules in healthy individuals and those with diabetes.

*In Conclusion*, the results are consistent with functional expression of SGLT2 after activation of CD4^+^ T cells, that enables concentrating glucose uptake into the cells, resulting in augmented release of inflammatory molecules like IFNγ. Importantly, inhibition of SGLT2 decreases IFNγ release.

## Data Availability

The raw data supporting the conclusions of this article will be made available by the authors, without undue reservation.
