# Supplementary material for: Inhibition of the Na+-glucose transporter SGLT2 reduces glucose uptake and IFNγ release from activated human CD4+ T cells
Source: Front Immunol. 2025 Jun 17;16:1576216. doi: 10.3389/fimmu.2025.1576216 (PMC12209339; doi:10.3389/fimmu.2025.1576216)
Supplement: Supplementary file 1 [file DataSheet1.pdf]

Supplementary information

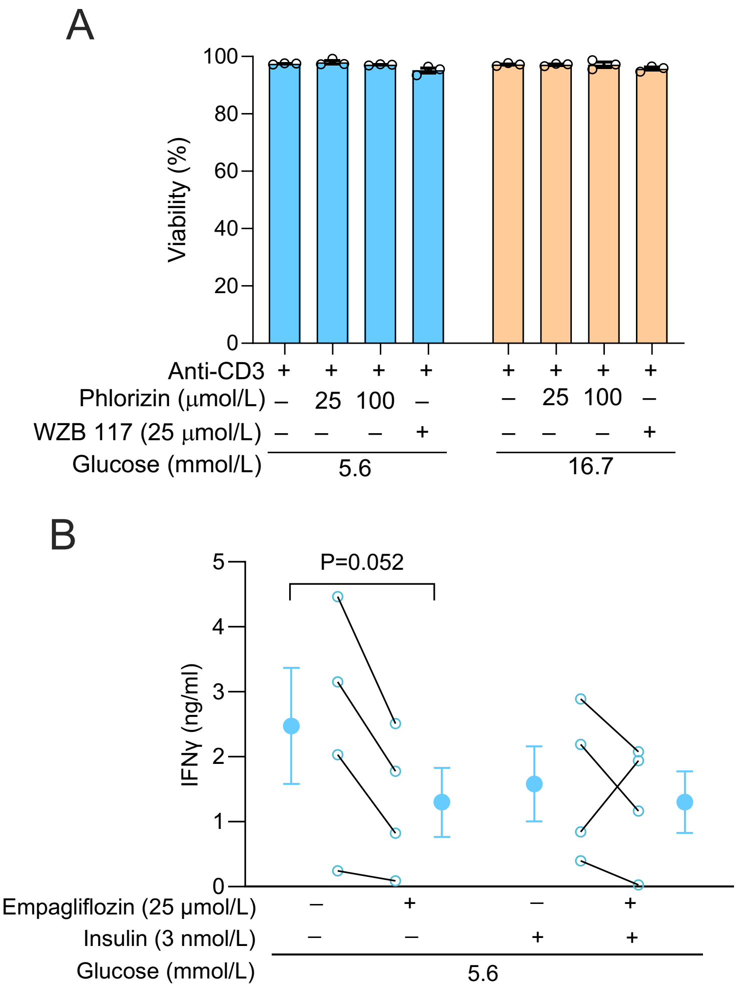

Supplementary Figure 1. The effect of SGLT and GLUT inhibitor on cell viability and IFNy release in activated human CD4⁺ T cells.

A) The cell viability of activated cells in presence of GLUT1 inhibitor, WZB 117 or SGLTs inhibitor, phlorizin in 5.6 mmol/L or 16.7 mmol/L glucose was measured with trypan blue exclusion test 72 h post-activation (N= 3). Data are presented mean ± SEM. **B)** IFNγ release from 72 h post-activated cells treated with or without empagliflozin (N=3) in the absence or presence of insulin, at 5.6 mmol/L glucose. Statistics: two-tailed paired Student's t-test. N: number of donors.
